# Supplementary material for: Dispersed emergence and protracted domestication of polyploid wheat uncovered by mosaic ancestral haploblock inference
Source: Nat Commun. 2022 Jul 6;13:3891. doi: 10.1038/s41467-022-31581-0 (PMC9259585; doi:10.1038/s41467-022-31581-0)
Supplement: Supplementary file 1 — Supplementary Information [file 41467_2022_31581_MOESM1_ESM.pdf]

Dispersed emergence and protracted domestication of polyploid wheat uncovered by mosaic ancestral haploblock inference

Wang *et al.*

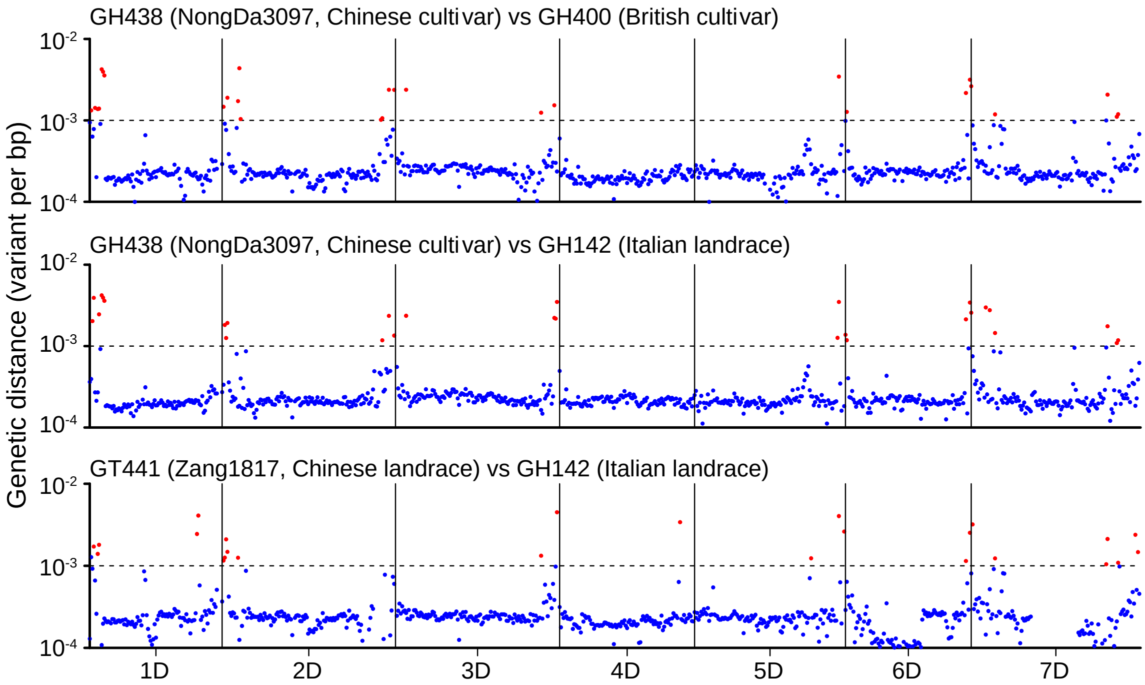

Supplementary Fig. 1. The binwise densities of pairwise genetic differences along 7 D chromosomes for the three accession pairs. Each point represents a 5 Mbp genomic window. Source data are provided as a Source Data file.

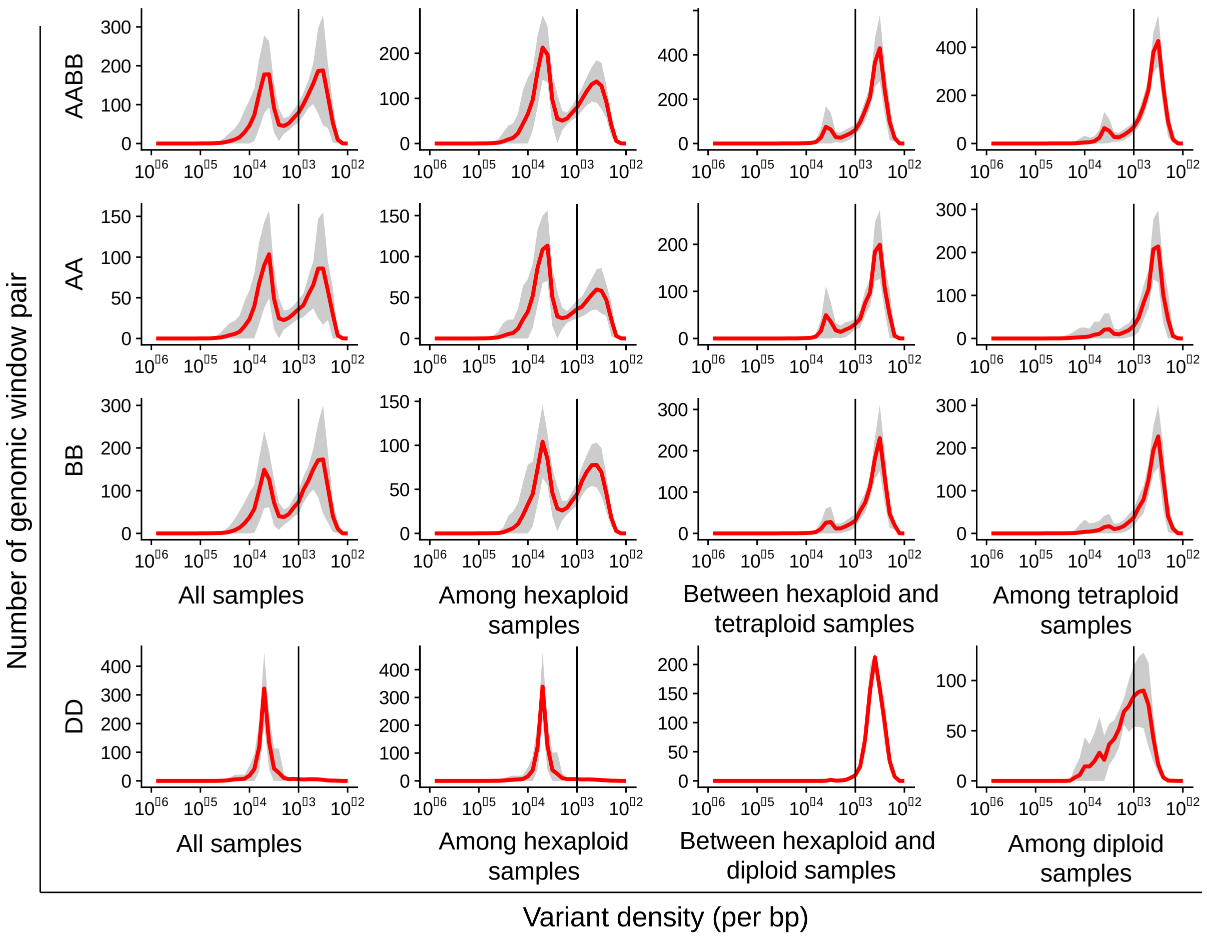

Supplementary Fig. 2. Distribution of pairwise genetic distances for 16 subgenome(s) and sample pair combinations. Solid red line and colored area, mean±sd. The black line indicates the 10^{-3} variants/bp threshold. 1000 sample pairs are randomly sampled for each profile except the D subgenome in diploid wheat. A total of 5 *Ae. tauschii* accessions were included in this study, and all 10 possible sample pairs were used. Source data are provided as a Source Data file.

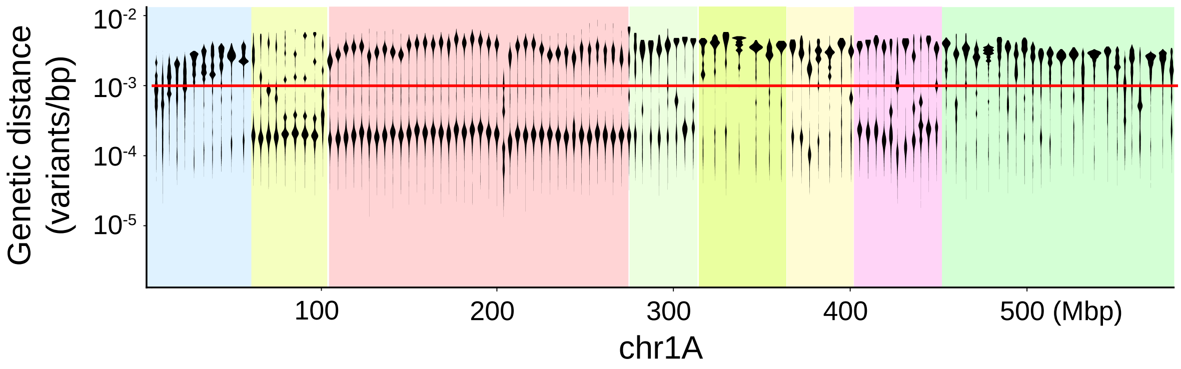

Supplementary Fig. 3. Distribution of genetic distance along chromosome 1A. Each column represents a 5 Mbp genomic window. Adjacent windows sharing similar distribution pattern are shaded by the same color. The red horizontal line indicated the 10^{-3} variants/bp threshold. Source data are provided as a Source Data file.

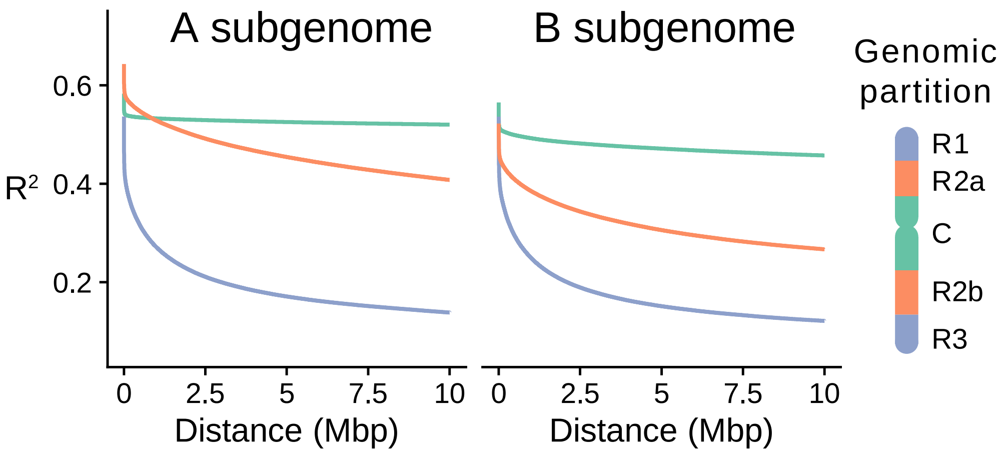

Supplementary Fig. 4. Decay of linkage disequilibrium (LD) in the A and B subgenome across 5 chromosomal zones. All tetraploid and hexaploid accessions were used for estimation. The chromosomal zones R1, R2a, C, R2b and R3 are consistent with Fig. 2a. Source data are provided as a Source Data file.

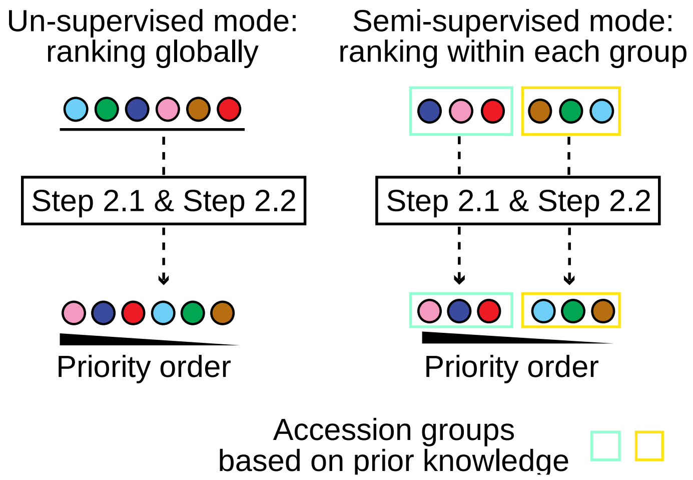

Supplementary Fig. 5. Schema of the un-supervised and the semi-supervised mode of the step 2 global AHG re-assignment. In un-supervised mode, the priority order of all accessions is adjusted globally. In semi-supervised mode, the adjustment is confined within each accession group assigned based on prior knowledge.

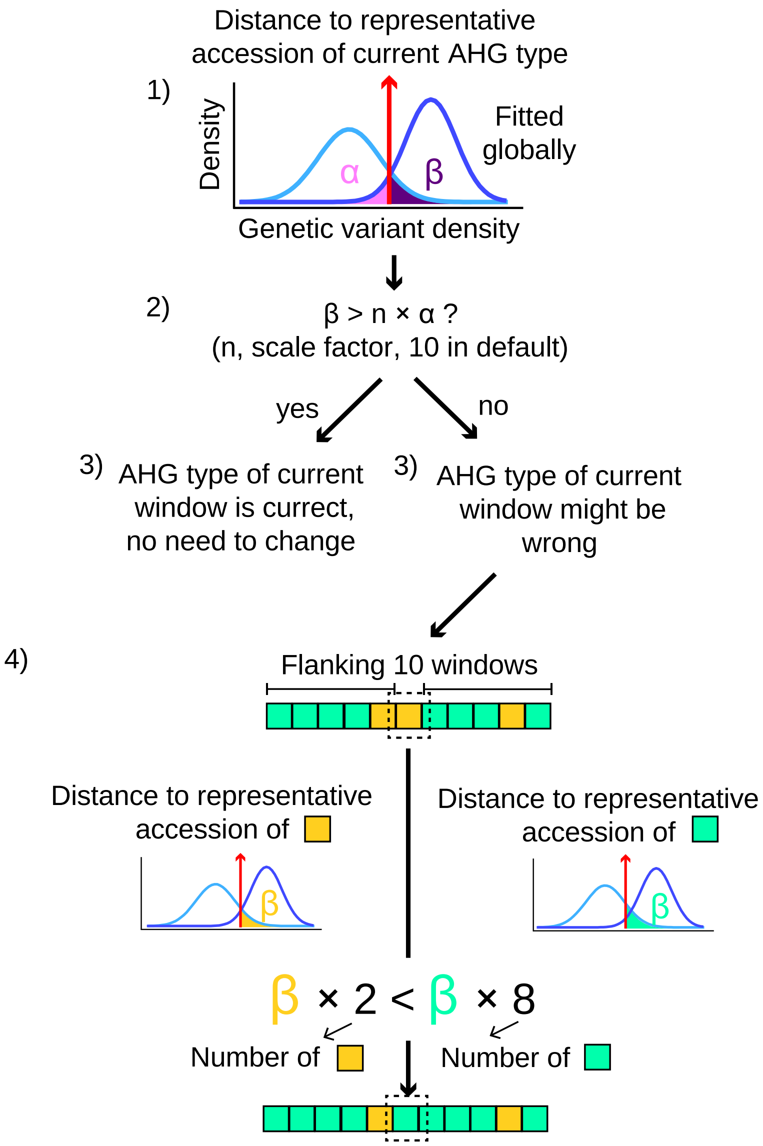

Supplementary Fig. 6. Schema of the step 3 Bayesian smoothing of IntroBlock algorithm. For each window of each accession, IntroBlocker first estimated the possibility of misassignment by the ratio between type I error (α) and type II error (β) of the current classification of AHG type. These two types of errors are quantified by the genetic distance to the representative sample Gaussian distributions components. If type II error (β) is greater than a given time (10 in default) of type I error (α), IntroBlocker renders this classification as correct and no change is needed. Otherwise, the possibility of all AHG types in flanking 10 windows is considered. IntroBlocker computed the product of the corresponding β value and number of windows for each AHG type, and the AHG changes to the type with maximum value.

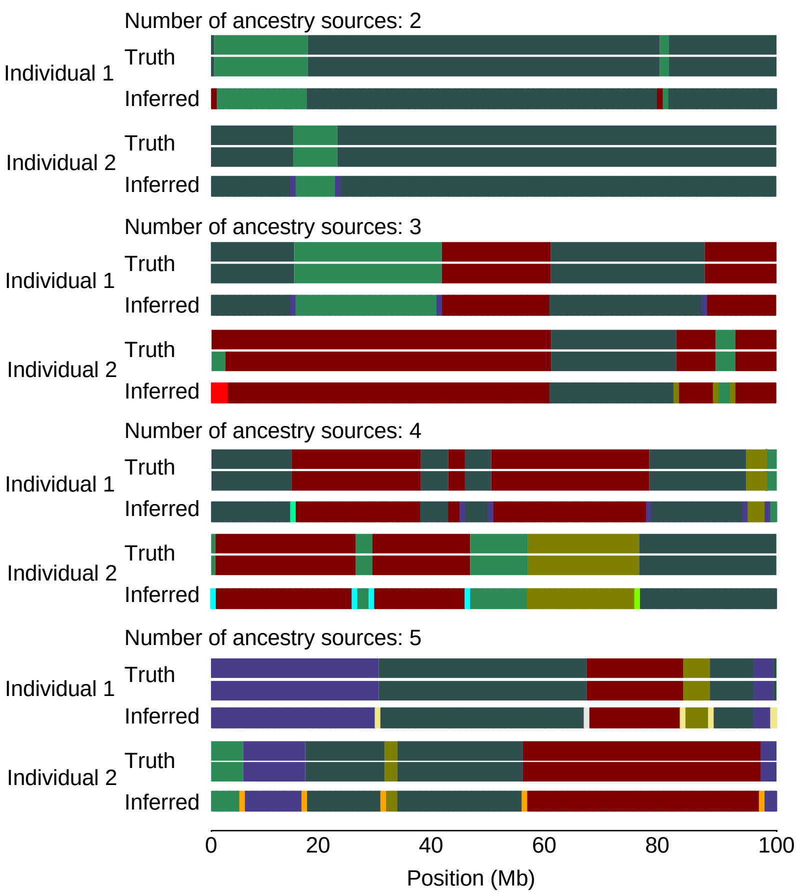

Supplementary Fig. 7. Simulated ancestries and corresponding inference using IntroBlocker along a 100Mb length pseudo-chromosome. Two individuals were shown under the scenario of 2~5 ancestry sources. Source data are provided as a Source Data file.

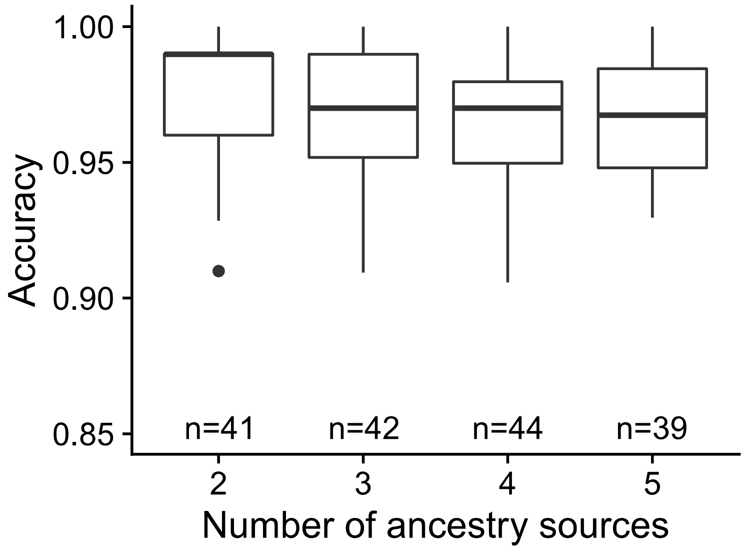

Supplementary Fig. 8. Inference accuracy of IntroBlocker under the scenario of 2~5 ancestry sources. 50 replications were conducted for each scenario. Individuals with heterozygous ratio>0.1 were excluded. The middle line indicates the median value. The lower and upper hinges correspond to the first and third quartiles (the 25th and 75th percentiles). The top whisker denotes the maximum value or the third quartile plus 1.5× the IQR, whichever is smaller. The bottom whisker denotes either the minimum value or the first quartile minus 1.5× the IQR, whichever is larger. Source data are provided as a Source Data file.

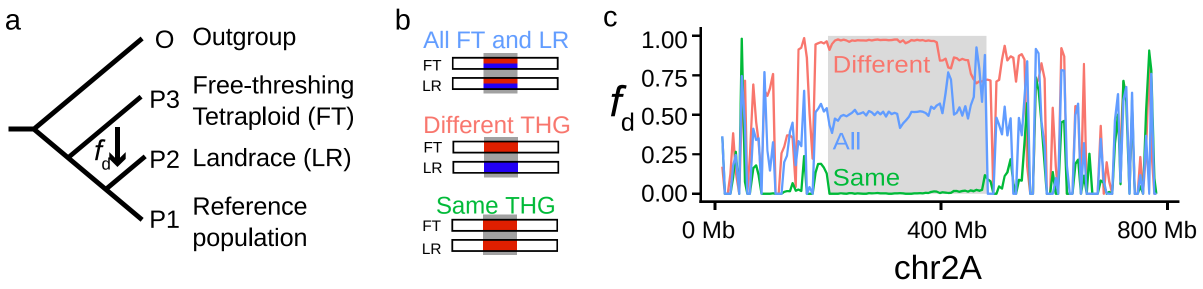

Supplementary Fig. 9. Case study of IntroBlocker algorithm superiority in introgression detection on chr2A. A large scale introgression signal was previously reported [1]. a, the four-taxon topology used for modeling introgression in ABBA–BABA tests. b, three strategies to select subset of FT and LR samples as the p2 and p3 population. Selecting all FT and LR samples regardless of their AHG type in the reported introgression region (indicated by shaded area). Blue and red rectangles indicated the two dominant AHG type detected in this region. c, the corresponding f_d distribution of three classification mentioned in (b) along the 2A chromosome. Source data are provided as a Source Data file.

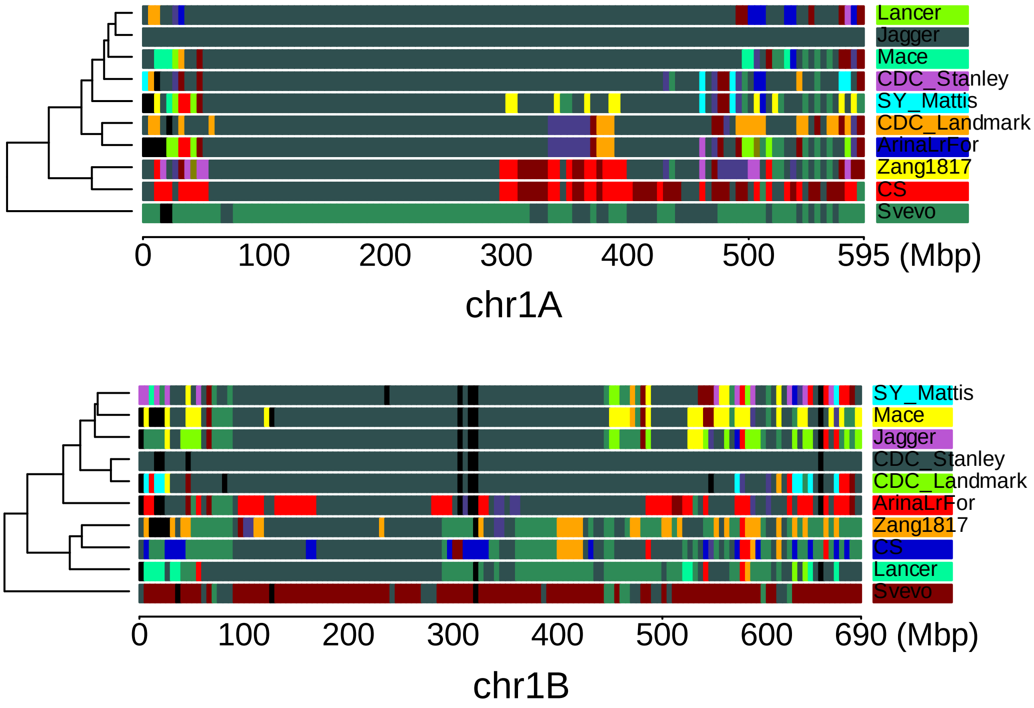

Supplementary Fig. 10. The colored mosaic graphs of AHGs in chromosome 1A and 1B across 10 wheat genome assemblies under the un-supervised mode. For each window, the same color indicates the same AHG and black indicates CNV block. The sample-dominated colors are marked under accession IDs. AHG-based phylogenetic tree is shown in the left. Source data are provided as a Source Data file.

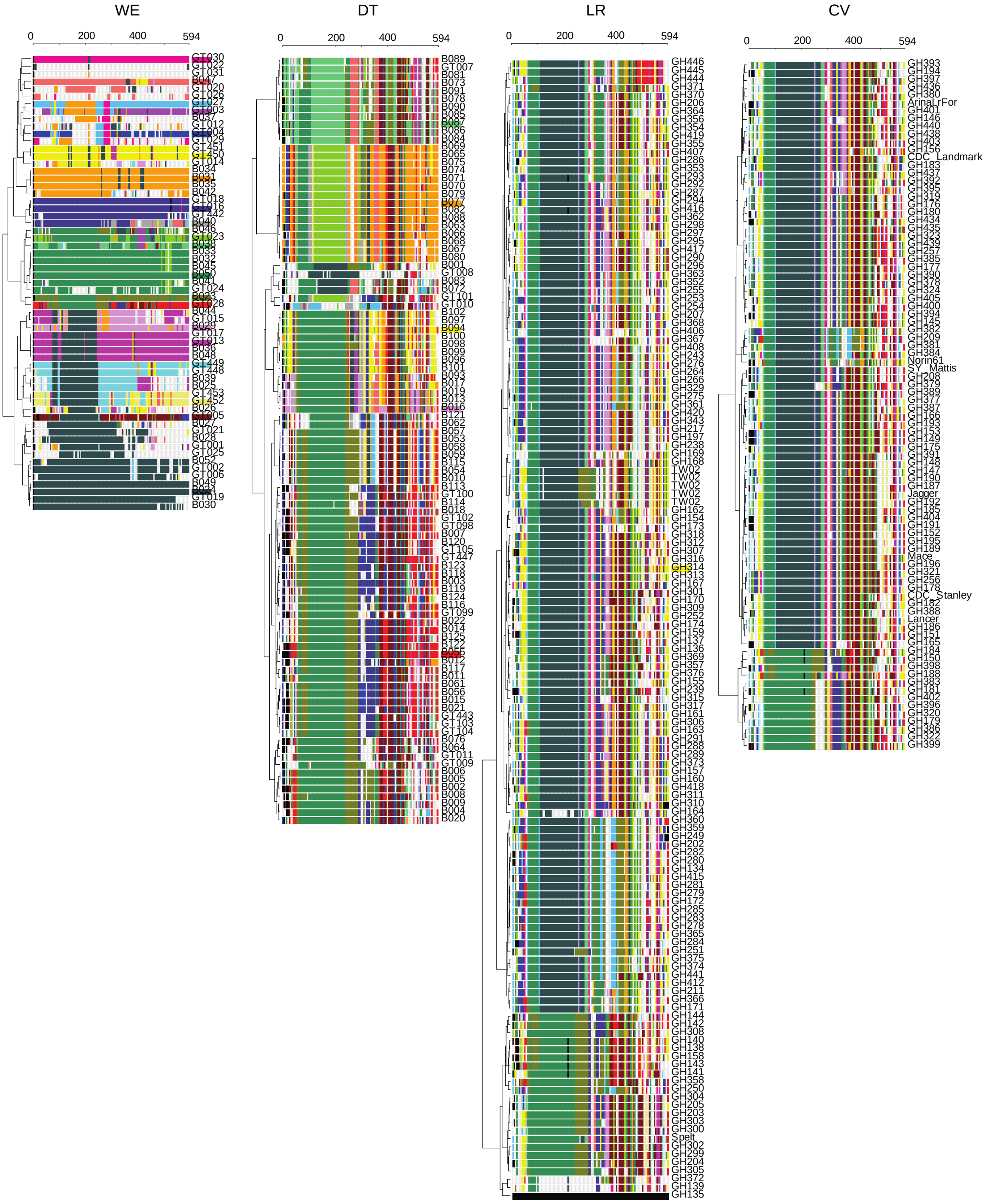

Supplementary Fig. 11. The mosaic views of AHG across chromosome 1A in semi-supervised mode with the priority of WE-DT-HW. For each window, the same color indicates the same AHG and black indicates CNV block. The sample-dominated colors are marked under accession-IDs. AHG-based phylogenetic tree is shown in the left to each of the four groups. WE, wild emmer wheat. DT, domesticated tetraploid wheat. LR, hexaploid landrace. CV, hexaploid cultivar wheat. Source data are provided as a Source Data file.

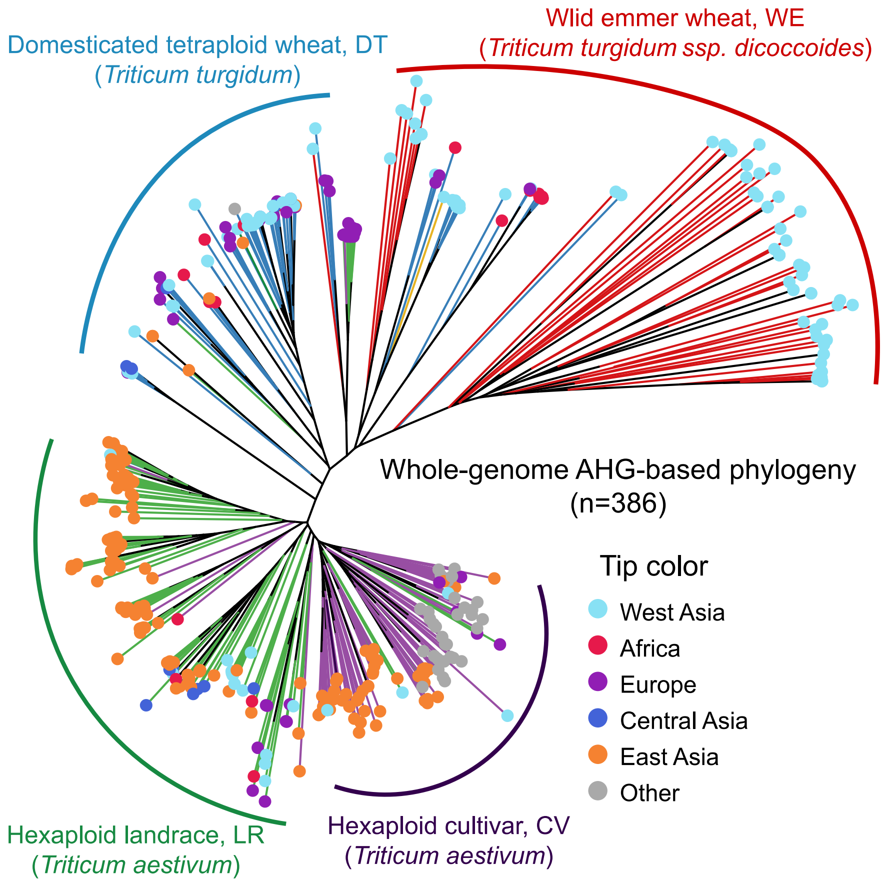

Supplementary Fig. 12. NJ-tree constructed with AHG-based distances for all 5-Mb windows in A&B genomes. Four major taxonomic groups are marked by colored lines along the circumference. Source data are provided as a Source Data file.

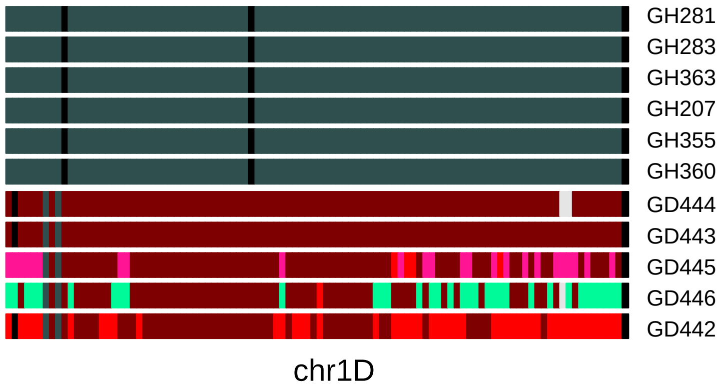

Supplementary Fig. 13. The mosaic views of AHG for selected accessions across chromosome 1D. For each window, the same color indicates the same AHG and black indicates CNV block. The sample-dominated colors are marked under accession-IDs. Source data are provided as a Source Data file.

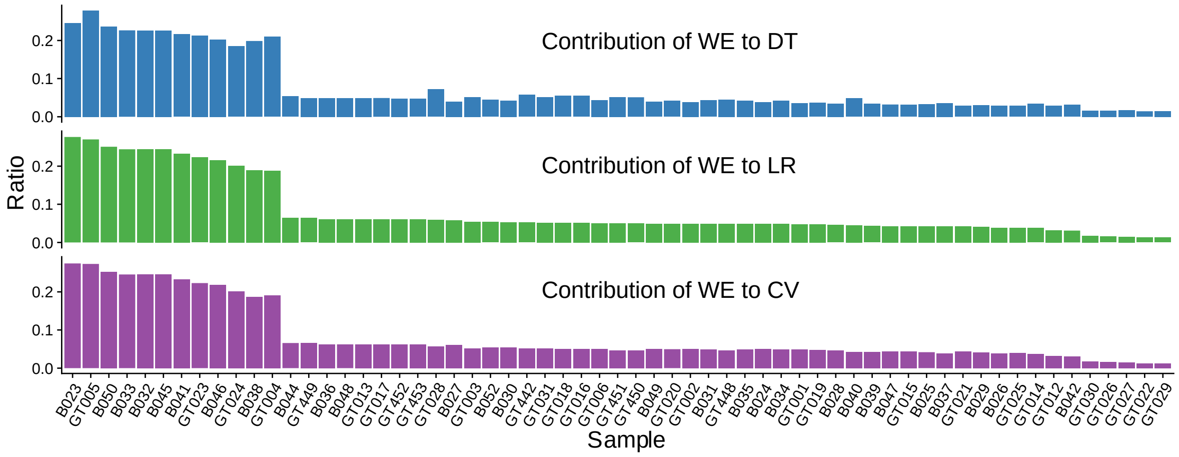

Supplementary Fig. 14. Genomic contributions of each WE accessions to DT (up), LR (middle) and CV (bottom). WE, wild emmer wheat. DT, domesticated tetraploid wheat. LR, hexaploid landrace. CV, hexaploid cultivar wheat. Source data are provided as a Source Data file.

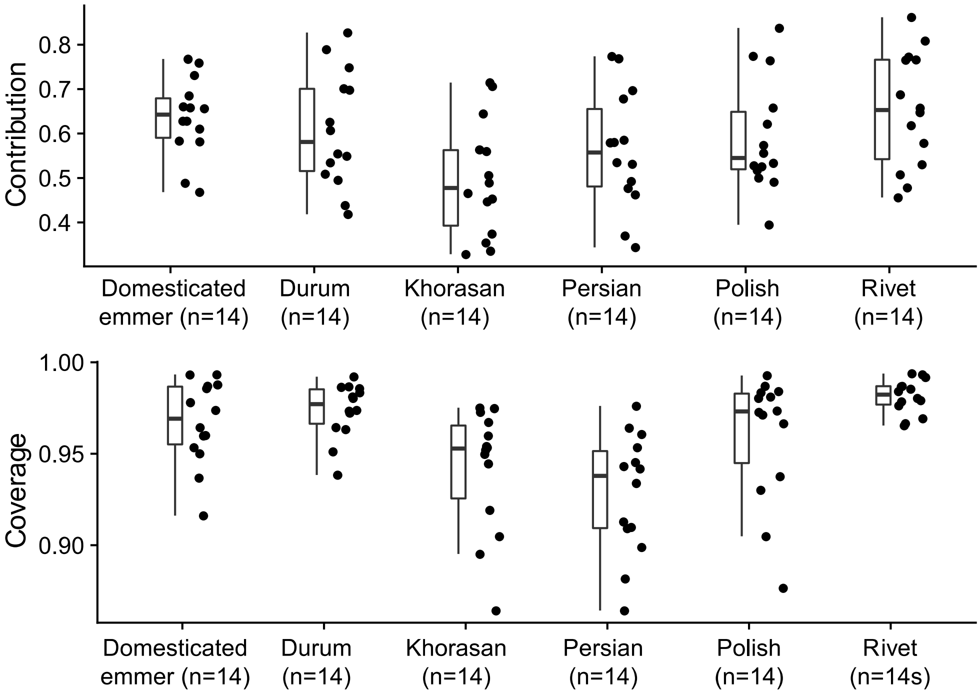

Supplementary Fig. 15. Genomic contributions and coverages of domesticated emmer wheat and each subspecies of free-threshing wheat to hexaploid landraces. Each point represents 1 of 14 chromosome in the A&B subgenomes. The middle line indicates the median value. The lower and upper hinges correspond to the first and third quartiles (the 25th and 75th percentiles). The top whisker denotes the maximum value or the third quartile plus 1.5× the IQR, whichever is smaller. The bottom whisker denotes either the minimum value or the first quartile minus 1.5× the IQR, whichever is larger. Source data are provided as a Source Data file.

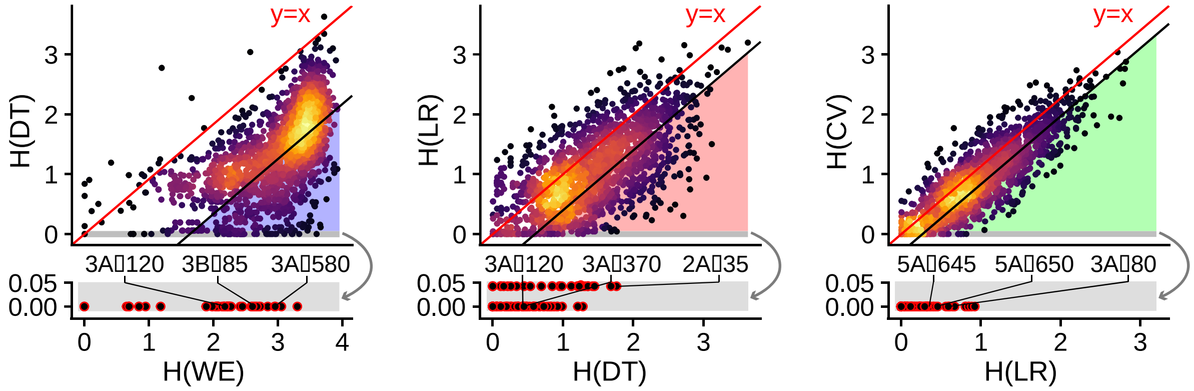

Supplementary Fig. 16. Relationship of the Shannon diversity index (H) of AHGs for all 5 Mbp windows (points) across A&B genomes between taxonomic groups. Signatures of selection were detected for windows with H_{WE}-H_{DT}>1.5, H_{DT}-H_{LR}>0.6 and H_{LR}-H_{CV}>0.3, which are indicated by colored areas. Windows with fixed AHGs (H<0.05) are noted in the enlarged panel. For example, 3A-120 indicates the window 3A:120-125 Mbp. WE, wild emmer wheat. DT, domesticated tetraploid wheat. LR, hexaploid wheat landrace. CV, hexaploid wheat cultivar. DE, domesticated emmer. DU, durum wheat. Source data are provided as a Source Data file.

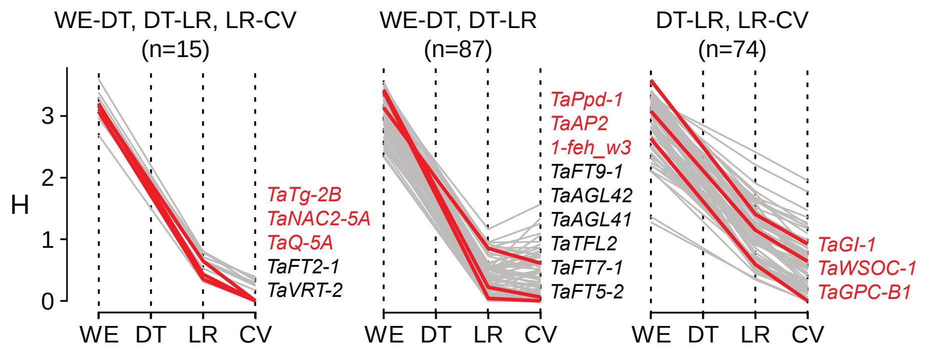

Supplementary Fig. 17. Dynamics in Shannon diversity index (H) for genomic windows under continuous selection in at least two stages. Each grey line represents a 5 Mbp window. Known adaptive genes residing in these windows are labeled. WE, wild emmer wheat. DT, domesticated tetraploid wheat. LR, hexaploid landrace. CV, hexaploid cultivar wheat. Source data are provided as a Source Data file.

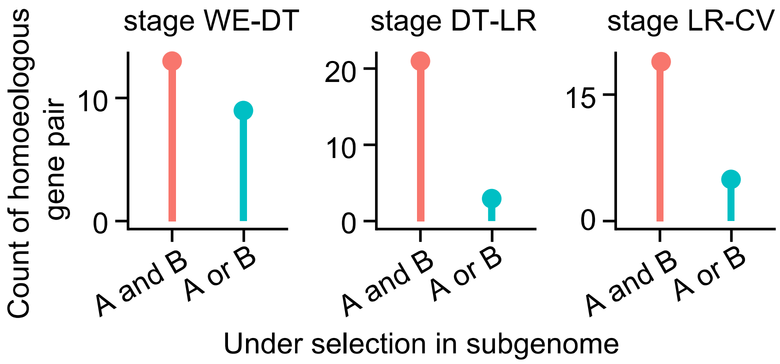

Supplementary Fig. 18. Count of known adaptive homoeologous gene pairs residing in genomic windows with selection signals detected in both A&B subgenomes (A and B), and in one of A or B subgenomes (A or B). WE, wild emmer wheat. DT, domesticated tetraploid wheat. LR, hexaploid landrace. CV, hexaploid cultivar wheat. Source data are provided as a Source Data file.

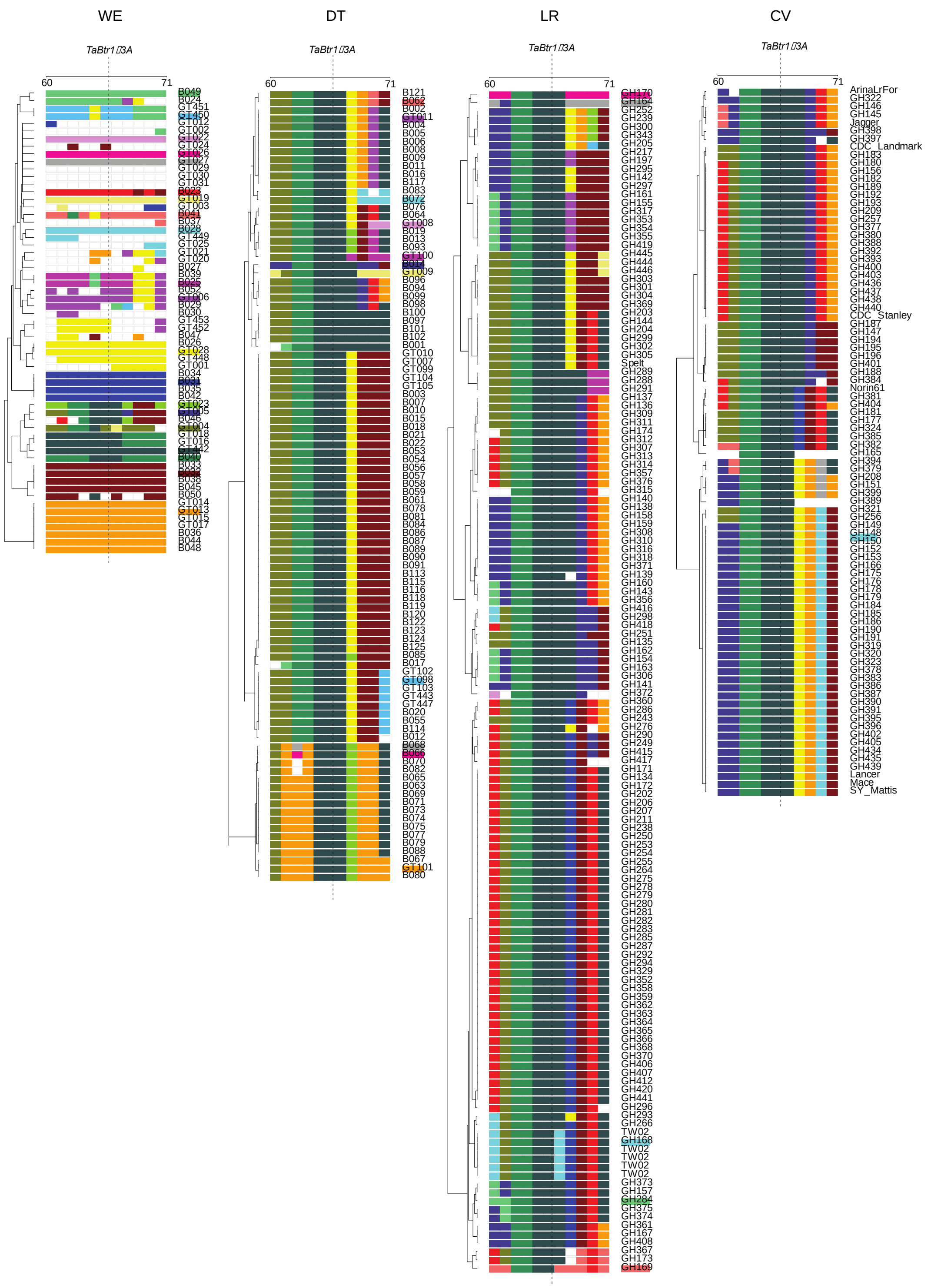

Supplementary Fig. 19. Colored mosaic graphs of AHGs around chr3A:65Mb where *TaBtr1-3A* located under semi-supervised mode with the priority of WE-DT-HW. AHG-based phylogenetic tree is shown in the left to each of the four groups. The exact location of *TaBtr1-3A* is indicated by the dash line. Each block represents a 1 Mbp genomic window. For each window, the same color indicates the same AHG. Black indicates CNV block. Grey indicates AHG types excess 20 in the priority order. The sample-dominated colors are marked under accession-IDs. WE, wild emmer wheat. DT, domesticated tetraploid wheat. LR, hexaploid landrace. CV, hexaploid cultivar wheat. Source data are provided as a Source Data file.

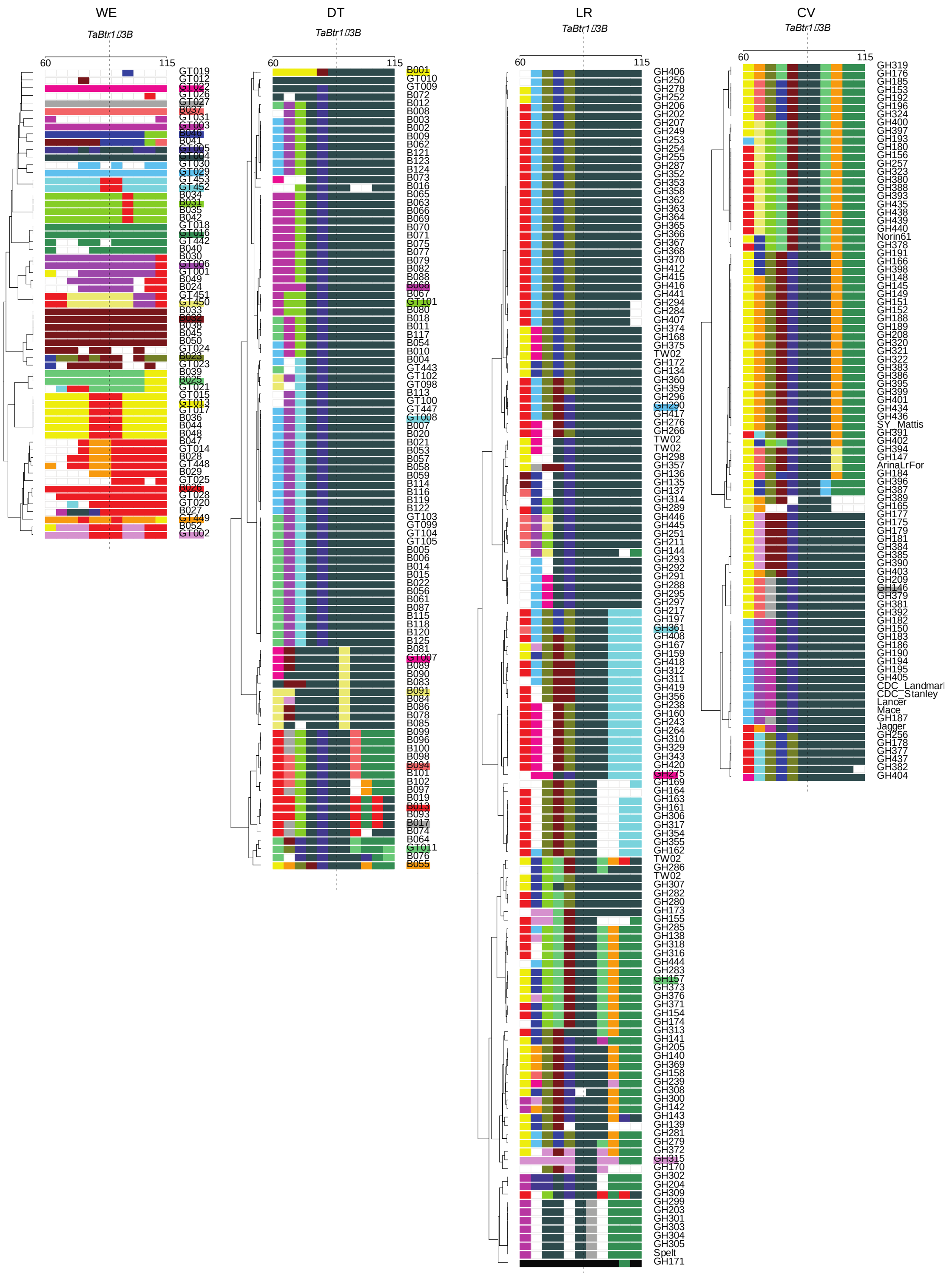

Supplementary Fig. 20. Colored mosaic graphs of AHGs around chr3B:89Mb where *TaBtr1-3B* located under semi-supervised mode with the priority of WE-DT-HW. AHG-based phylogenetic tree is shown in the left to each of the four groups. The exact location of *TaBtr1-3B* is indicated by the dash line. Each block represents a 5 Mbp genomic window. For each window, the same color indicates the same AHG. Black indicates CNV block. Grey indicates AHG types excess 20 in the priority order. The sample-dominated colors are marked under accession-IDs. WE, wild emmer wheat. DT, domesticated tetraploid wheat. LR, hexaploid landrace. CV, hexaploid cultivar wheat. Source data are provided as a Source Data file.

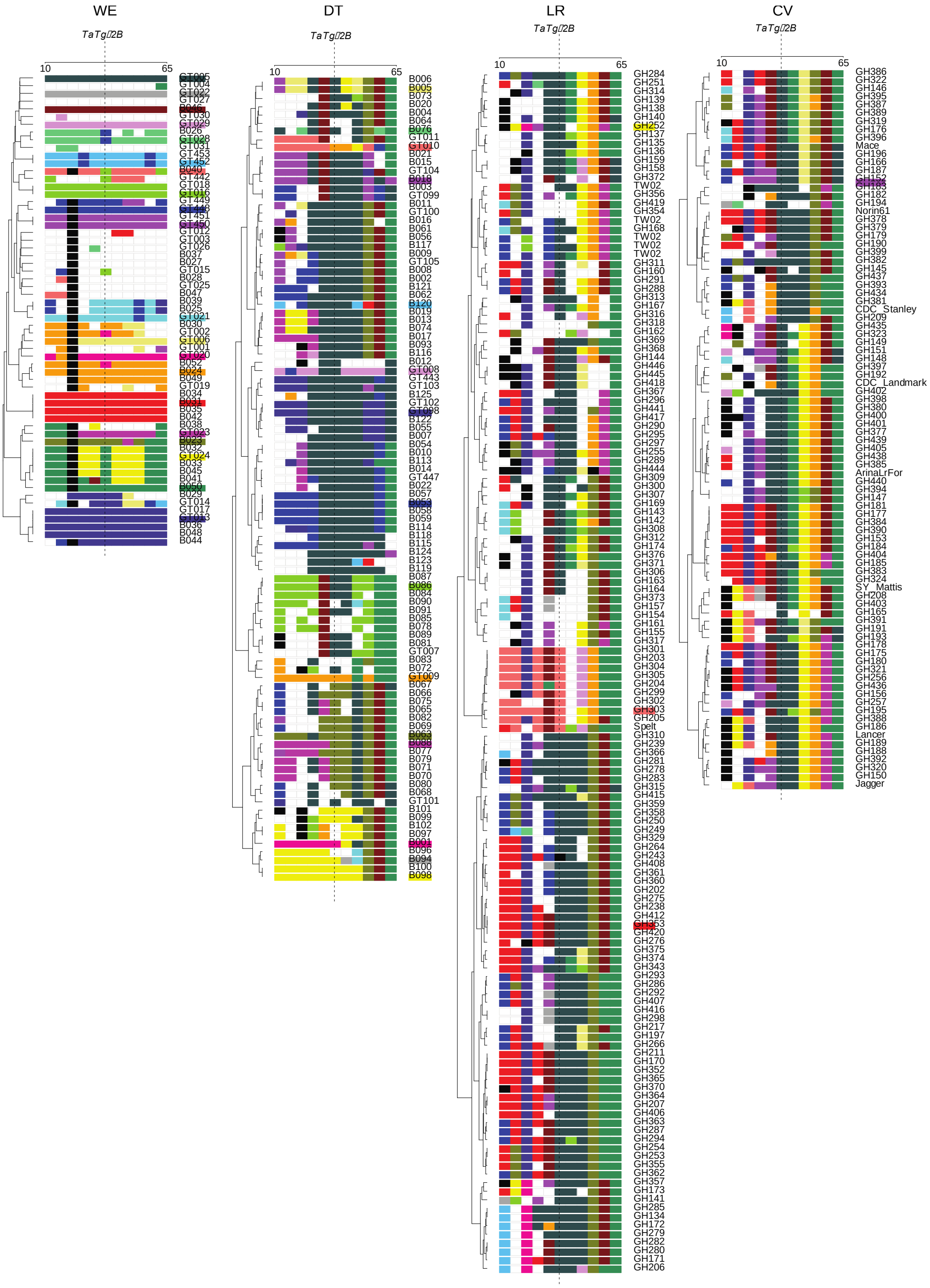

Supplementary Fig. 21. Colored mosaic graphs of AHGs around chr2B:37Mb where *TaTg1-2B* located under semi-supervised mode with the priority of WE-DT-HW. AHG-based phylogenetic tree is shown in the left to each of the four groups. The exact location of *TaTg1-2B* is indicated by the dash line. Each block represents a 5 Mbp genomic window. For each window, the same color indicates the same AHG. Black indicates CNV block. Grey indicates AHG types excess 20 in the priority order. The sample-dominated colors are marked under accession-IDs. WE, wild emmer wheat. DT, domesticated tetraploid wheat. LR, hexaploid landrace. CV, hexaploid cultivar wheat. Source data are provided as a Source Data file.

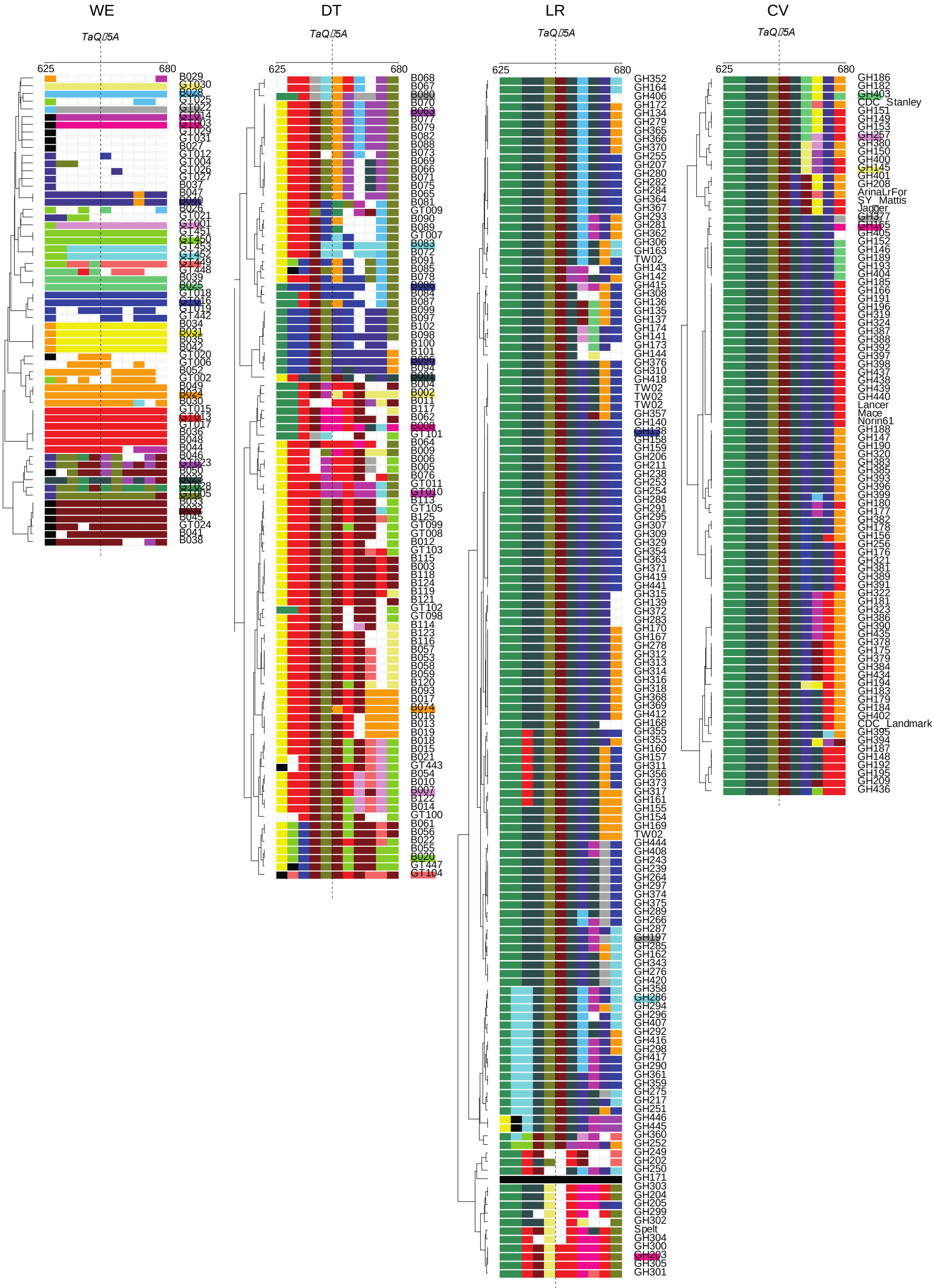

Supplementary Fig. 22. Colored mosaic graphs of AHGs around chr3A:650Mb where *TaQ-5A* located under semi-supervised mode with the priority of WE-DT-HW. AHG-based phylogenetic tree is shown in the left to each of the four groups. The exact location of *TaQ-5A* is indicated by the dash line. Each block represents a 5 Mbp genomic window. For each window, the same color indicates the same AHG. Black indicates CNV block. Grey indicates AHG types excess 20 in the priority order. The sample-dominated colors are marked under accession-IDs. WE, wild emmer wheat. DT, domesticated tetraploid wheat. LR, hexaploid landrace. CV, hexaploid cultivar wheat. Source data are provided as a Source Data file.

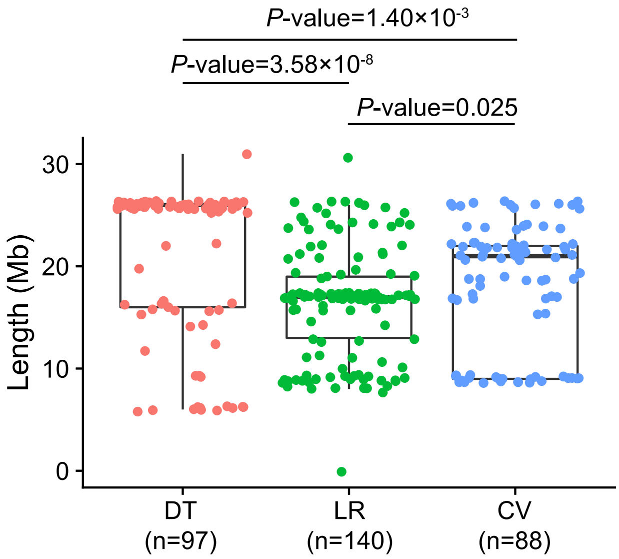

Supplementary Fig. 23. Length distribution of continues windows around *TaBtr1-3B* under fixation. *P*-values are based on two-tailed *t*-test. DT, domesticated tetraploid wheat. LR, hexaploid landrace. CV, hexaploid cultivar wheat. The middle line indicates the median value. The lower and upper hinges correspond to the first and third quartiles (the 25th and 75th percentiles). The top whisker denotes the maximum value or the third quartile plus 1.5× the IQR, whichever is smaller. The bottom whisker denotes either the minimum value or the first quartile minus 1.5× the IQR, whichever is larger. Source data are provided as a Source Data file.

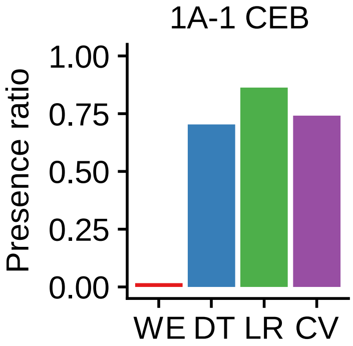

Supplementary Fig. 24. Frequency of the presence of 1A-1 CEB in four taxonomic groups. WE, wild emmer wheat. DT, domesticated tetraploid wheat. LR, hexaploid wheat landrace. CV, hexaploid wheat cultivar. Source data are provided as a Source Data file.

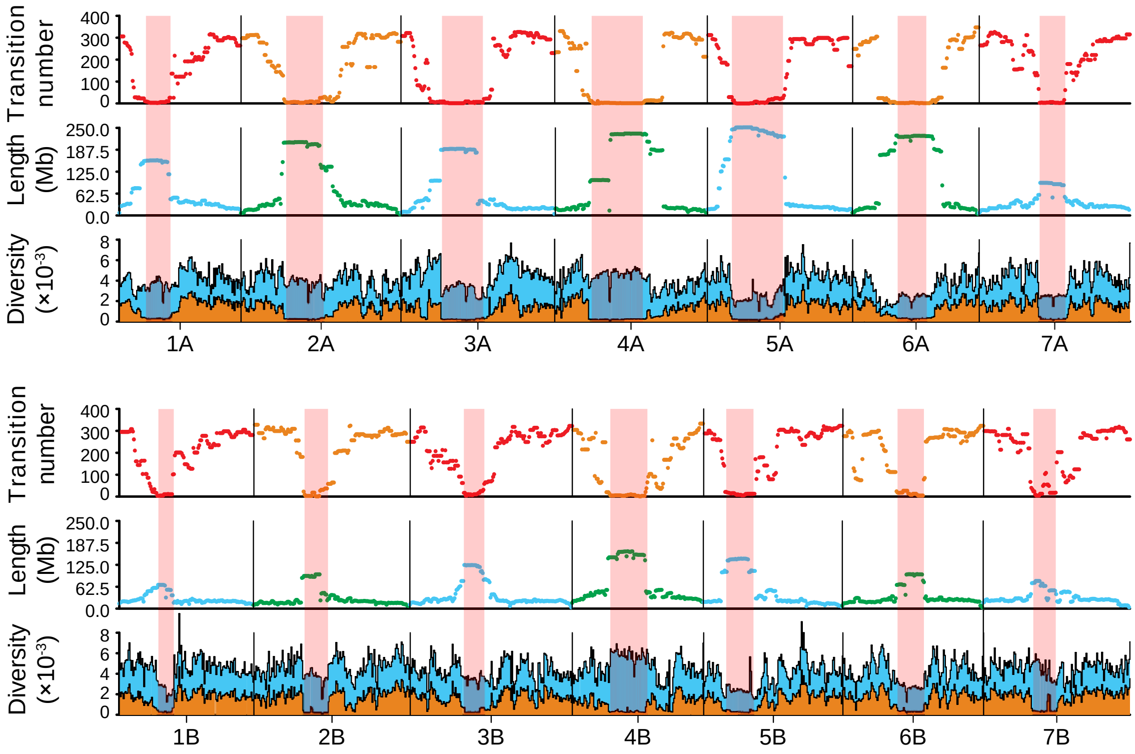

Supplementary Fig. 25. Positioning of centromeric ancestral haplotype group (centAHG) in 14 A&B chromosomes. It is based on time of transition among AHG types between adjacent windows (upper), length of consecutive windows of same type of AHG (middle) and ratio between inter- and intra- AHG type genetic diversity (lower) of all 386 accessions. Color area indicates centAHG. Source data are provided as a Source Data file.

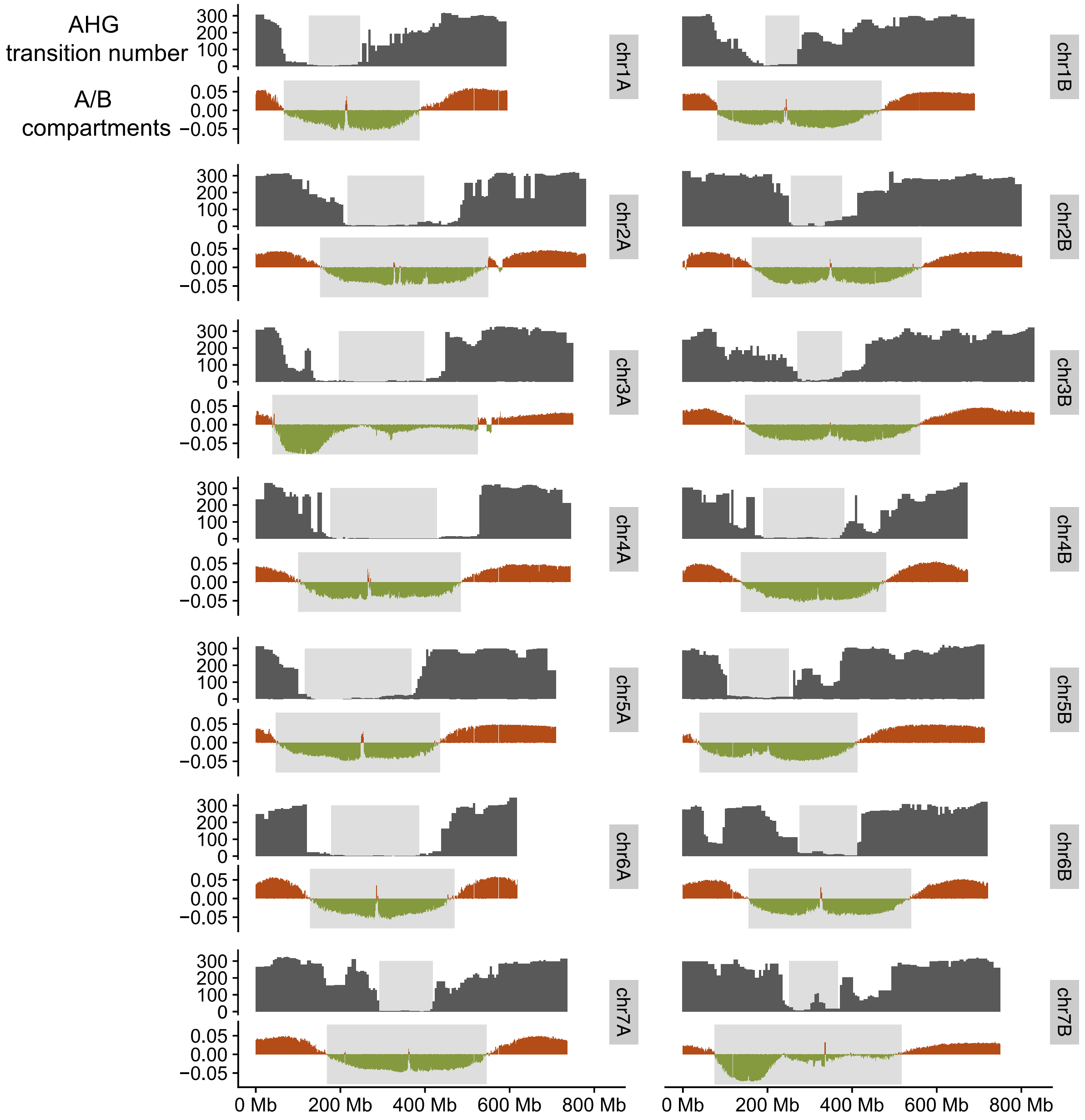

Supplementary Fig. 26. The consistency between the partition of the centAHG and the A/B compartment from a previously published study[2] on 14 chromosomes in A&B subgenome.

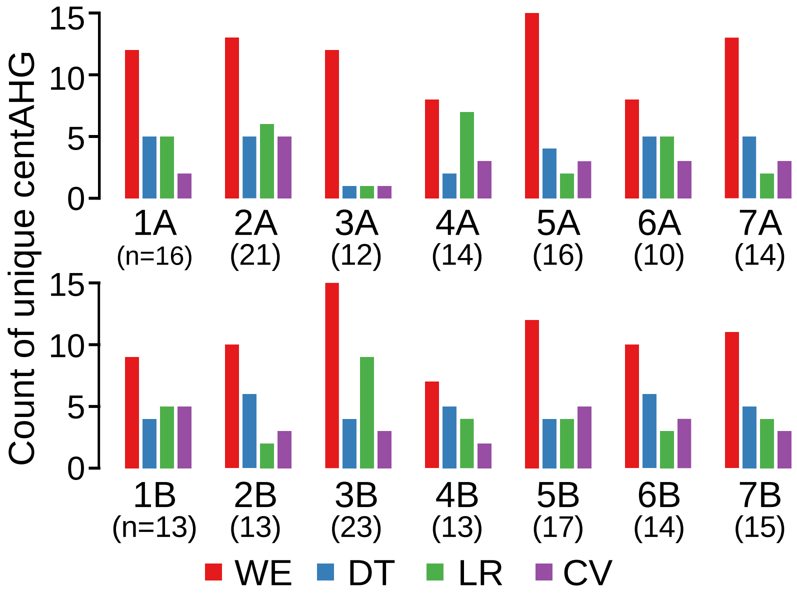

Supplementary Fig. 27. Counts of unique centAHGs in the four groups. Source data are provided as a Source Data file.

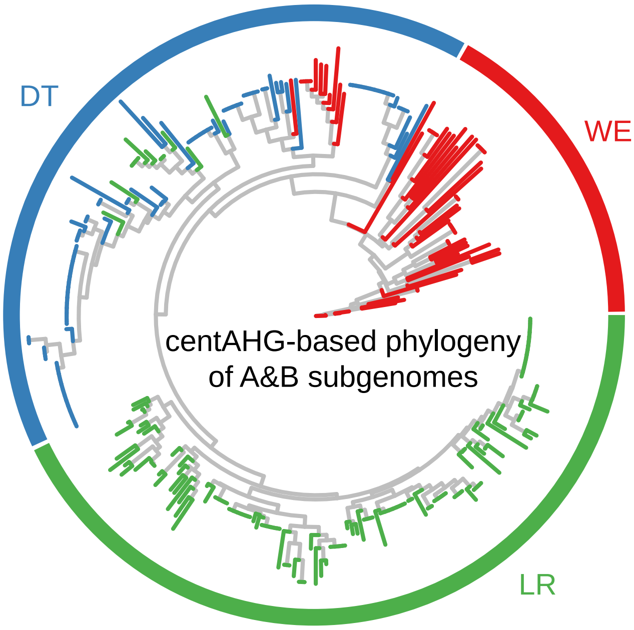

Supplementary Fig. 28. PAH-based neighbor-joining phylogenetic tree for A&B subgenomes. Major taxonomic groups are marked by colored lines along the circumference. Source data are provided as a Source Data file.

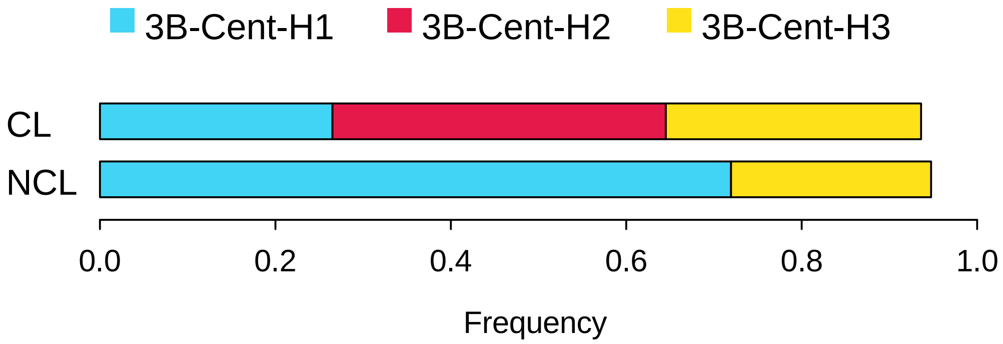

Supplementary Fig. 29. Frequency of main 3B centAHGs in landraces. Whole genome resequencing refers to this study. CL: Chinese Landrace. NCL: Non-Chinese Landrace. Source data are provided as a Source Data file.

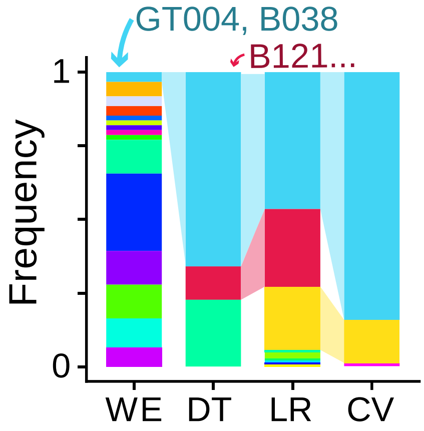

Supplementary Fig. 30. Frequency of centAHG-3B types in four groups. 3 main centAHG-3B types are connected through groups with colored ribbon. WE accessions carrying 3B-Cent-H1 and DT accessions carrying 3B-Cen-H2 were labeled with IDs. Due to space limit, the accession list for 3B-Cen-H2 is incomplete. The full list: B001, B062, B094, B096, B097, B098, B099, B100, B101, B102, B121. WE, wild emmer wheat. DT, domesticated tetraploid wheat. LR, hexaploid landrace. CV, hexaploid cultivar wheat. Source data are provided as a Source Data file.

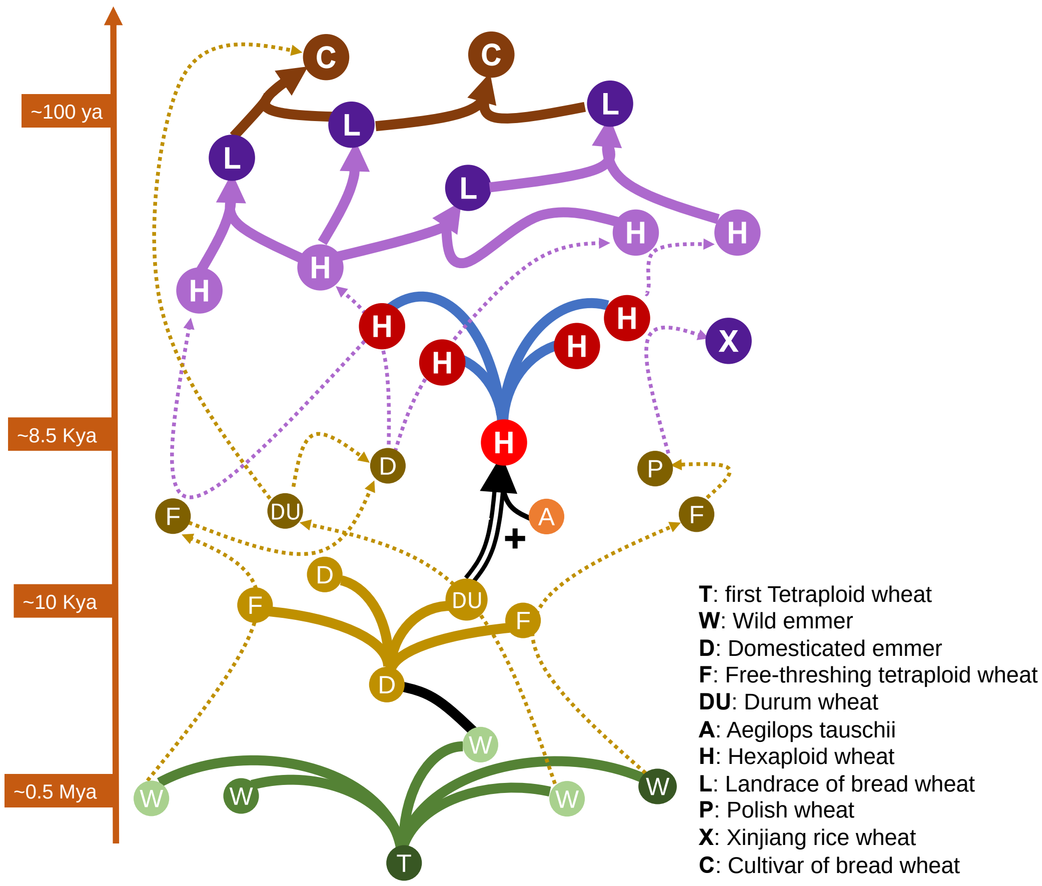

Supplementary Fig. 31. The evolutionary model of nested origin and tangled domestication process of tetraploid and hexaploid wheat with corresponding time scale. Each point represent a derived lineage of corresponding species. The solid line represents the relationship of direct progeny. The dotted line represents hybridization event starting from the the point of paternal line, through maternal line, and ending at their offspring. The black arrow and the plus sign represents the hybridization event that led to the birth of hexaploid wheat. This schema is based on results in this study and prior assumptions from the literature.

Supplementary Table 1. Detailed sequencing information of the 16 accessions newly obtained in this study.

Sample	Raw data (bp)	Clean data (bp)	Average depth
GT001	85,712,727,600	84,442,354,800	5.73
GT002	62,568,060,000	61,405,463,100	4.33
GT003	80,008,516,800	78,958,406,400	5.5
GT004	131,553,148,200	129,606,600,900	8.16
GT005	81,446,201,700	79,834,756,800	4.99
GT006	72,153,879,900	70,929,372,900	4.54
GT007	72,011,680,200	70,842,673,200	4.68
GT008	77,544,744,300	76,304,340,300	4.99
GT009	74,076,585,600	72,979,995,000	5.23
GT010	68,278,996,500	67,295,043,600	4.71
GT011	63,910,438,800	62,823,009,300	4.53
GT103	74,626,465,200	74,217,551,700	5.39
GT104	115,539,460,800	113,278,823,100	7.18
GT105	75,076,333,200	73,967,868,300	5.29
GW01	112,016,559,300	110,443,152,300	1.84
GW02	110,610,771,300	109,074,279,000	1.82

Supplementary Table 2. Means and SDs of the fitted Gaussian distribution and estimated divergence time.

Profile	SD	Mean	Time	Mean-SD	Time	Mean +SD	Time
DD genome of HW	0.36	2.04	5261.40	1.68	6359.90	2.46	7687.74
AABB genome of HW	1.05	2.02	6308.22	0.96	3018.08	4.22	13185.09
AABB genome of DT	1.10	2.27	7078.43	1.17	3671.20	4.37	13647.87

*HW: hexaploid wheat; DT: domesticated emmer; SD and mean: $\times 10^{-4}$ variants/bp; Time: years.

Supplementary Table 3. Percentage of windows with different AHG inheritance pattern in A&B subgenomes.

WE	DT	LR	CV	Count	Percentage (%)
NO	NO	NO	YES	3496	0.45
NO	NO	YES	NO	12362	1.57
NO	NO	YES	YES	33198	4.23
NO	YES	NO	NO	27035	3.44
NO	YES	NO	YES	4555	0.58
NO	YES	YES	NO	17126	2.18
NO	YES	YES	YES	153715	19.57
YES	NO	NO	NO	91697	11.67
YES	NO	NO	YES	1140	0.15
YES	NO	YES	NO	905	0.12
YES	NO	YES	YES	8257	1.05
YES	YES	NO	NO	16749	2.13
YES	YES	NO	YES	3633	0.46
YES	YES	YES	NO	14628	1.86
YES	YES	YES	YES	397501	50.60

*YES: The AHG type was found in corresponding taxonomic group; NO: The AHG type was NOT found in corresponding taxonomic group

Supplementary Table 4. Positioning of centAHG for the A&B subgenomes.

Chr	Start (Mb)	End (Mb)
chr1A	130	250
chr1B	200	280
chr2A	220	400
chr2B	260	380
chr3A	200	400
chr3B	275	380
chr4A	180	430
chr4B	195	385
chr5A	120	370
chr5B	115	255
chr6A	220	360
chr6B	280	415
chr7A	295	420
chr7B	255	370

Supplementary references

1 Yuan, J. *et al.* Open chromatin interaction maps reveal functional regulatory elements and chromatin architecture variations during wheat evolution. *Genome Biol* **23**, 34 (2022).

2 Zhou, Y. *et al.* *Triticum* population sequencing provides insights into wheat adaptation. *Nat Genet* **52**, 1412-1422 (2020).
