# Supplementary material for: Dispersed emergence and protracted domestication of polyploid wheat uncovered by mosaic ancestral haploblock inference
Source: Nat Commun. 2022 Jul 6;13:3891. doi: 10.1038/s41467-022-31581-0 (PMC9259585; doi:10.1038/s41467-022-31581-0)
Supplement: Supplementary file 3 — Description of Additional Supplementary Files [file 41467_2022_31581_MOESM3_ESM.pdf]

Description of Additional Supplementary Files

File Name: Supplementary Data 1
Description: Passport information of whole-genome resequencing accessions in this study.

File Name: Supplementary Data 2
Description: AHG type of all 5 Mbp windows for 386 accessions in the A&B subgenomes.

File Name: Supplementary Data 3
Description: AHG type of all 5 Mbp windows for 313 accessions in D subgenomes.

File Name: Supplementary Data 4
Description: Genotype of chloroplast genome for 306 accessions.

File Name: Supplementary Data 5
Description: Windows with signature of selection detected in more than one evolutionary stages.

File Name: Supplementary Data 6
Description: Windows with signature of fixation detected.

File Name: Supplementary Data 7
Description: Frequency of presence in four taxonomic groups for 46 CEB.

File Name: Supplementary Data 8
Description: Presence and absence of the predominant AHG type around domesticationrelated and adaptation-related genes.

File Name: Supplementary Data 9
Description: centAHG type of 386 accessions for 14 A&B chromosomes.

File Name: Supplementary Data 10
Description: Inferred centAHG-3B type of additional 240 hexaploid landrace accessions.
