# Supplementary material for: Dispersed emergence and protracted domestication of polyploid wheat uncovered by mosaic ancestral haploblock inference
Source: Nat Commun. 2022 Jul 6;13:3891. doi: 10.1038/s41467-022-31581-0 (PMC9259585; doi:10.1038/s41467-022-31581-0)
Supplement: Supplementary file 14 — Reporting Summary [file 41467_2022_31581_MOESM14_ESM.pdf]

nature portfolio

Corresponding author(s): Qixin Sun, Weilong Guo

Last updated by author(s): Jun 16, 2022

Reporting Summary

Nature Portfolio wishes to improve the reproducibility of the work that we publish. This form provides structure for consistency and transparency in reporting. For further information on Nature Portfolio policies, see our Editorial Policies and the Editorial Policy Checklist.

Statistics

For all statistical analyses, confirm that the following items are present in the figure legend, table legend, main text, or Methods section.

n/a	Confirmed	
☐	☒	The exact sample size (*n*) for each experimental group/condition, given as a discrete number and unit of measurement
☐	☒	A statement on whether measurements were taken from distinct samples or whether the same sample was measured repeatedly
☐	☒	The statistical test(s) used AND whether they are one- or two-sided<br>*Only common tests should be described solely by name; describe more complex techniques in the Methods section.*
☐	☒	A description of all covariates tested
☐	☒	A description of any assumptions or corrections, such as tests of normality and adjustment for multiple comparisons
☐	☒	A full description of the statistical parameters including central tendency (e.g. means) or other basic estimates (e.g. regression coefficient) AND variation (e.g. standard deviation) or associated estimates of uncertainty (e.g. confidence intervals)
☐	☒	For null hypothesis testing, the test statistic (e.g. *F*, *t*, *r*) with confidence intervals, effect sizes, degrees of freedom and *P* value noted<br>*Give P values as exact values whenever suitable.*
☒	☐	For Bayesian analysis, information on the choice of priors and Markov chain Monte Carlo settings
☒	☐	For hierarchical and complex designs, identification of the appropriate level for tests and full reporting of outcomes
☒	☐	Estimates of effect sizes (e.g. Cohen's *d*, Pearson's *r*), indicating how they were calculated

Our web collection on statistics for biologists contains articles on many of the points above.

Software and code

Policy information about availability of computer code

Data collection	No software was used for data collection.
Data analysis	Softwares used in this study include the followings: Trimmomatic(v0.36), Bamtools(v2.4.1), Samtools(v1.3.1), GATK(v3.8), PLINK(v1.9), BWA-mem(v0.7.15), bedtools(v2.27.1), SnpEff(v4.3), SLiM(v3.7), iTOL(v6), Python(v3.7.12), Python module pySLiM(v0.700) and msprime(v1.1.0), script ABBABABAwindows.py from https://github.com/simonhmartin/genomics_general, R language(v3.6.0), R package mixtools(v1.2.0) and ape(v5.3), IntroBlocker(v1)[https://wangzihell.github.io/IntroBlocker]. The scripts developed in the study have been deposited at [https://github.com/wangzihell/CAU-MosaicWheat].

For manuscripts utilizing custom algorithms or software that are central to the research but not yet described in published literature, software must be made available to editors and reviewers. We strongly encourage code deposition in a community repository (e.g. GitHub). See the Nature Portfolio guidelines for submitting code & software for further information.

Data

Policy information about availability of data

All manuscripts must include a data availability statement. This statement should provide the following information, where applicable:

- Accession codes, unique identifiers, or web links for publicly available datasets
- A description of any restrictions on data availability
- For clinical datasets or third party data, please ensure that the statement adheres to our policy

The raw sequence data generated in this study have been deposited in the Sequence Read Archive under accession code PRJNA759292 [https://www.ncbi.nlm.nih.gov/bioproject/PRJNA759292/]. The raw sequence data generated in this study have also been deposited in the Genome Sequence Archive under accession code PRJCA006360 [https://ngdc.cncb.ac.cn/bioproject/browse/PRJCA006360]. The raw sequence data of previously published re-sequenced accessions

used in this study are available in the Sequence Read Archive under accession code PRJNA476679 [https://www.ncbi.nlm.nih.gov/bioproject/PRJNA476679], PRJNA596843 [https://www.ncbi.nlm.nih.gov/bioproject/PRJNA596843], PRJNA544491 [https://www.ncbi.nlm.nih.gov/bioproject/PRJNA544491], PRJNA492239 [https://www.ncbi.nlm.nih.gov/bioproject/PRJNA492239], PRJNA528431 [https://www.ncbi.nlm.nih.gov/bioproject/PRJNA528431], PRJNA544491 [https://www.ncbi.nlm.nih.gov/bioproject/PRJNA544491], PRJNA439156 [https://www.ncbi.nlm.nih.gov/bioproject/PRJNA439156], PRJNA663409 [https://www.ncbi.nlm.nih.gov/bioproject/PRJNA663409] and PRJEB22687 [https://www.ncbi.nlm.nih.gov/bioproject/PRJEB22687]. The raw sequence data of previously published re-sequenced accessions used in this study are available in the Genome Sequence Archive (http://bigd.big.ac.cn/gsa) under accession number CRA003763 [https://ngdc.cncb.ac.cn/gsa/browse/CRA003763] and CRA001873 [https://ngdc.cncb.ac.cn/gsa/browse/CRA001873]. The whole-exome capture dataset is available at http://wheatgenomics.plantpath.ksu,edu/1000EC/. The Chinese Spring wheat reference genome (IWGSC RefSeq v1.0) is publicly available at [https://wheat-urgi.versailles.inra.fr/Seq-Repository/Assemblies].

Field-specific reporting

Please select the one below that is the best fit for your research. If you are not sure, read the appropriate sections before making your selection.

☐ Life sciences ☐ Behavioural & social sciences ☒ Ecological, evolutionary & environmental sciences

For a reference copy of the document with all sections, see nature.com/documents/nr-reporting-summary-flat.pdf

Ecological, evolutionary & environmental sciences study design

All studies must disclose on these points even when the disclosure is negative.

Study description	We generated a pan-ancestry map by applying an ancestral genomic introgression block dissection method, IntroBlocker, to a resequencing dataset of 386 tetraploid/hexaploid wheat accessions.
Research sample	We collected the whole genome re-sequencing data of a panel of 393 interploidy wheat accessions comprising 7 diploid accessions (5 Aegilops tauschii with DD genome and 2 Aegilops longissima with SS genome), 158 tetraploid accessions (Triticum turgidum with BBAA genome) and 228 worldwide hexaploid accessions (Triticum aestivum with BBAADD genome). This collection was selected to represent the diversity of evolutionary stages and geographic distribution of polyploid wheat.
Sampling strategy	No sample size calculated was performed. We collected as many wheat whole genome re-sequencing data publicly available as we could and we sequenced as many accessions as we could afford. This diverse collection covered the major evolutionary stages of polyploid wheat, including wild emmer wheat, domesticated emmer wheat, free threshing tetraploid wheat, etc.
Data collection	Whole-genome resequencing data of 16 accessions were generated in this study, and the other 377 accessions were collected from a total of 8 sources. Genomic DNA was extracted from young leaves of 16 accessions following a standard CTAB protocol by Sun group. DNA libraries were constructed by Novogene and sequenced with the Illumina Hiseq Xten PE150 platform. The data collected and generated in this study were analysed together.
Timing and spatial scale	Samples were planted and sequenced at the seedling stage in 2018.
Data exclusions	No data excluded.
Reproducibility	Not directly applicable, as this study focused on characterizing the existing patterns of genetic diversity and there is no experiments involved. All the computational replications were included in the results.
Randomization	Randomization does not directly apply to the characterization of genetic diversity. However, it does apply to some of the computational analyses conducted. Details of these cases are described in the methods and adhere to widely accepted standards. For example, 1000 accession pairs were randomly chosen to characterize the genetic distance distribution (e.g. Fig. 1b).
Blinding	Not applicable. This study focuses on genomic data of plant and no analyses required being blind to groups.

Did the study involve field work? ☐ Yes ☒ No

Reporting for specific materials, systems and methods

We require information from authors about some types of materials, experimental systems and methods used in many studies. Here, indicate whether each material, system or method listed is relevant to your study. If you are not sure if a list item applies to your research, read the appropriate section before selecting a response.

Materials & experimental systems

n/a	Involved in the study
☒	☐ Antibodies
☒	☐ Eukaryotic cell lines
☒	☐ Palaeontology and archaeology
☒	☐ Animals and other organisms
☒	☐ Human research participants
☒	☐ Clinical data
☒	☐ Dual use research of concern

Methods

n/a	Involved in the study
☒	☐ ChIP-seq
☒	☐ Flow cytometry
☒	☐ MRI-based neuroimaging
